# Factors Associated with Major Versus Minor Lower Extremity Amputation in Hospitalized Patients with Diabetes in Saudi Arabia

**DOI:** 10.3390/jcm15207946

**Published:** 2026-10-14

**Authors:** Fathia Ahmed Mersal, Salma M. Alzarawi, Nahed A. Mersal, Ahmed Hazazi, Motab Aljohani, Sahal Alzahrani

**Affiliations:** 1 College of Nursing, Northern Border University, Arar 73312, Saudi Arabia; 2 Medical Surgical Nursing Department, Al Noor Specialist Hospital, Makkah 24242, Saudi Arabia; salzarawi@moh.gov.sa; 3 College of Nursing, King Abdulaziz University, Jeddah 21589, Saudi Arabia; namali@kau.edu.sa; 4 Department of Public Health, College of Health Sciences, Saudi Electronic University, Riyadh 13316, Saudi Arabia; a.hazazi@seu.edu.sa (A.H.); m.aljohani@seu.edu.sa (M.A.); s.alzahrani@seu.edu.sa (S.A.)

**Keywords:** diabetic foot, lower extremity amputation, peripheral neuropathy, ischemic heart disease, peripheral arterial disease, risk factors

## Abstract

**Background**: Major lower extremity amputation (LEA) is a severe complication of diabetes with substantial disability and mortality. Evidence from Saudi Arabia with distinguishing factors linked to major versus minor amputation is limited. We examined demographic, clinical, vascular, and wound-related factors associated with amputation level among hospitalized patients with diabetes who had already undergone LEA. **Methods**: We conducted a retrospective comparative observational study of 230 adults with diabetes who underwent non-traumatic LEA at Al Noor Specialist Hospital, Makkah. Because the cohort comprised exclusively patients who had already undergone amputation, the analysis was explanatory in purpose and characterized factors associated with the level of amputation (major versus minor) rather than predictors of amputation occurrence in the broader population of patients with diabetic foot disease. Independent associations were examined using multivariable binary logistic regression with Firth’s penalization; adjusted odds ratios (aORs) with 95% confidence intervals were the principal results, and exploratory model diagnostics. **Results**: Major amputation occurred in 92 patients (40.0%); 138 (60.0%) underwent minor amputation. Mean age was 58.4 ± 12.6 years; 77.0% were male. After adjustment, ischemic heart disease (adjusted odds ratio [aOR] 5.20, 95% confidence interval [CI] 1.63–16.55), multiple foot ulcers (aOR 4.65, 95% CI 1.53–14.17), ulcer duration >1 month (aOR 3.15, 95% CI 1.09–9.10), and documented peripheral neuropathy (aOR 7.46, 95% CI 1.13–49.35) were independently associated with major amputation. The neuropathy estimate was imprecise, reflecting the small number of documented cases, and should therefore be regarded as highly uncertain. These exploratory associations describe the conditional comparison of amputation level within this operated cohort and should not be interpreted as individual probabilities of amputation or amputation level. **Conclusions**: Ischemic heart disease, multiple ulcers, prolonged ulcer duration, and documented peripheral neuropathy were associated with major amputation. These associations should be interpreted within the limits of a retrospective, single-center design with possible underascertainment of neuropathy and peripheral arterial disease. These exploratory findings require replication in independent, prospectively designed cohorts before they can be generalized to other settings or applied clinically.

## 1. Introduction

Diabetes mellitus (DM) is a major global public health challenge. The International Diabetes Federation estimated that 537 million adults were living with diabetes in 2021, with a continuing increase projected over subsequent decades [1]. Diabetic foot ulcers (DFU) and lower extremity amputation (LEA) are among the most consequential lower-extremity complications of diabetes because they are associated with recurrent care needs, loss of mobility, impaired quality of life, and considerable mortality [2,3,4,5]. These outcomes arise along a clinical continuum, but ulcer development, progression to any amputation, and selection of a major rather than minor amputation represent distinct outcomes that should not be treated as interchangeable.

Saudi Arabia has a substantial diabetes burden, increasing the number of people potentially exposed to long-term microvascular and macrovascular complications [1,6]. Hospital-based diabetic foot disease also carries important resource implications within the Saudi health system [7]. Longitudinal evidence from another national health system shows that patterns of diabetic foot complications and LEA can change over time, underscoring the importance of setting-specific evidence [8]. Nevertheless, regional studies have more often described diabetes prevalence, diabetic foot burden, or amputation occurrence than the factors that distinguish a proximal major procedure from a distal minor procedure among patients who have already reached operative limb loss. This distinction is clinically important because the two procedure levels differ in functional consequences, rehabilitation requirements, and the intensity of post-discharge support.

Diabetic limb loss commonly develops through interacting neuropathic, vascular, infectious, and wound-related pathways [8,9]. Loss of protective sensation permits repetitive trauma to remain unnoticed, while peripheral arterial disease (PAD) reduces tissue perfusion and constrains healing [9,10,11]. Infection and the anatomic extent of tissue loss may further limit the feasibility of a distal operation. Systemic cardiovascular comorbidity can also identify patients with a broader atherosclerotic burden. Ischemic heart disease (IHD), in particular, may coexist with lower-extremity arterial disease and may therefore be relevant to the level of amputation among patients who already require surgical treatment [11,12].

The level at which an amputation is performed is not determined by a single biological process. It reflects the distribution of viable tissue, the extent of necrosis or infection, the capacity for a wound to heal, the available arterial supply, and the clinical feasibility of preserving a functional distal segment. These domains can be correlated in an advanced diabetic foot presentation. Consequently, an association between one recorded characteristic and amputation level may partly represent information shared with other measured or unmeasured aspects of limb threat. An explanatory analysis must therefore report adjusted estimates while avoiding claims that an individual factor, in isolation, determined the surgical level.

The distinction between major and minor LEA also defines a specific research question. Studies that compare patients who undergo any amputation with those managed without amputation estimate factors related to amputation occurrence. By contrast, a study restricted to amputated patients compares procedure level within an operative subgroup. Reviews and hospital-based studies have documented factors associated with LEA in diabetic foot populations [4], but those findings cannot be transferred automatically to a conditional comparison of major versus minor procedures. A focused analysis of amputation level can complement, rather than replace, research on ulcer prevention and limb salvage.

The clinical setting in Makkah adds a relevant referral context. Al Noor Specialist Hospital provides tertiary services to residents, expatriates, and pilgrims and receives patients with complex diabetic foot presentations. The present data do not quantify referral delay, prior treatment, or access barriers, so those pathways cannot be assigned a direction or magnitude in this analysis. The setting should instead be understood as a heterogeneous tertiary-care environment in which documented cardiovascular disease, neurovascular status, and wound burden may vary substantially at the time of admission. Evidence from the region supports the importance of diabetic foot risk factors and the healthcare burden of advanced ulcer care, while also showing why locally generated analyses require careful definition of their source populations [7,13].

A more detailed characterization of this conditional outcome may assist clinicians and researchers in identifying which information should be documented consistently when patients present with advanced disease. Cardiovascular history, standardized sensory assessment, objective perfusion testing, wound number, wound duration, infection severity, and prior treatment may each contribute different information. The present retrospective dataset cannot reconstruct every element of that pathway, but it can clarify which documented characteristics were associated with a more proximal procedure in this hospital cohort and identify the measurements that prospective studies should collect more systematically.

Because major amputation is linked to greater disability, higher mortality, and higher resource utilization than distal procedures [14], clarifying factors associated with major versus minor LEA is clinically relevant. The present study aimed to (1) describe the distribution of major and minor amputation in a tertiary-care cohort of patients who had already undergone LEA, (2) examine clinical and wound-related factors associated with amputation level among patients who had already undergone amputation, and (3) report supplementary exploratory diagnostics of the fitted multivariable model (discrimination and calibration statistics) in the Supplementary Materials. Because the cohort consisted exclusively of patients who had already undergone amputation, the study identifies characteristics associated with a more proximal amputation level rather than predictors of amputation in the broader population of patients with DFU. The analysis was therefore explanatory in orientation, the reported associations are exploratory, and no individual probability of amputation or of amputation level is estimated.

## 2. Methods

### 2.1. Ethical Statement

Ethical approval was obtained from the Institutional Review Board of the Ministry of Health, Saudi Arabia (Reference: IRB-2022-045-SA). The study was conducted in accordance with the Declaration of Helsinki and reported with reference to the Strengthening the Reporting of Observational Studies in Epidemiology (STROBE) statement [15]. The requirement for individual informed consent was waived by the Institutional Review Board because only de-identified routinely collected clinical data were analyzed.

### 2.2. Study Design and Setting

This retrospective observational study was conducted at Al Noor Specialist Hospital, a 500-bed tertiary academic medical center in Makkah, Saudi Arabia. The hospital serves as a referral center for complex diabetic foot disease and provides vascular surgery, orthopedic, endocrinology, and inpatient medical services. Eligible patients who underwent lower extremity amputation between 1 January 2017 and 31 December 2020, were identified through hospital records. Because the analytic framework was restricted to patients who had already undergone lower extremity amputation, the study compares major versus minor amputation and does not estimate the probability of amputation among the wider population of patients with diabetic foot disease. The analysis was designed to be explanatory rather than predictive: the multivariable model characterizes the associations of clinical, vascular, and wound-related factors with amputation level among amputated patients, and does not estimate the probability of amputation occurrence.

The analysis aimed to assess the association of each prespecified characteristic with the odds of major versus minor amputation among eligible patients undergoing LEA at the study hospital during the 2017–2020 period. The source population was therefore defined by hospitalization, diabetes, a non-traumatic indication, and completion of an amputation procedure. This conditioned study base describes variation in surgical level within the observed operative case mix. It does not supply a denominator for estimating DFU incidence, LEA incidence, or the probability that a patient with DFU will progress to surgery.

### 2.3. Participants

Eligible participants were adults (≥18 years) with type 1 or type 2 diabetes mellitus who underwent a non-traumatic lower extremity amputation at Al Noor Specialist Hospital during the study period. Potential cases were identified through the hospital surgical registry and electronic medical record system using ICD-10 procedure codes for lower limb amputation. Inclusion criteria were age ≥ 18 years, documented diabetes mellitus according to American Diabetes Association criteria, and admission for a major or minor non-traumatic LEA. Exclusion criteria were traumatic amputation unrelated to diabetic complications, amputation for malignancy, incomplete records lacking essential predictor or outcome data, and transfer from an outside facility solely for postoperative care. A total of 258 potential cases were screened. After exclusion of 12 trauma-related amputations, 4 tumor-related amputations, and 12 records with substantial missing data, 230 patients were included in the final cohort (Figure S1). To ensure a one-patient-one-amputation principle, only the index amputation procedure was included for patients with multiple or staged procedures; for bilateral amputations, the highest level of amputation was recorded. Major amputation was defined as amputation at or proximal to the ankle joint, including ankle disarticulation and all more proximal procedures (below-knee, through-knee, and above-knee amputations); minor amputation was defined as amputation distal to the ankle joint, including toe, ray, and transmetatarsal procedures as recorded in the source record. “Previous foot ulcer” was defined as any documented history of a diabetic foot ulcer prior to the current admission, and “previous amputation” was defined as any history of minor or major lower extremity amputation prior to the index procedure. With 92 patients in the major amputation group and six model terms, the ratio of outcome events to model parameters was approximately 15.3, supporting reasonably stable multivariable estimation.

Cohort assembly proceeded from the 258 potentially eligible procedures identified in the registry and medical record system. Exclusion of 12 trauma-related cases and 4 tumor-related cases aligned the cohort with the non-traumatic diabetic-foot question. A further 12 records were excluded because essential outcome or explanatory-variable information was substantially incomplete, leaving 230 participants for analysis. This approach produced an analyzable cohort but may have reduced representativeness if excluded records differed systematically from retained records. The handling of repeat, staged, bilateral, or multiple procedures was confirmed to align with the aforementioned rules established in the source protocol.

### 2.4. Data Collection and Study Variables

Data were abstracted between May and November 2022 by trained research assistants using a standardized pilot-tested form. Information was obtained from electronic medical records, nursing documentation, operative reports, discharge summaries, and the laboratory information system. A random 10% sample of records was re-abstracted by a senior investigator, with inter-rater agreement exceeding 95%. Variable-level missingness was low, ranging from 0.0% to 4.8% (Supplementary Table S1); therefore, complete-case analysis was used, and the missing-data matrix is presented in Supplementary Table S1. The full cohort of 230 patients was used for all descriptive statistics and bivariate analyses. The multivariable model was fitted on the complete-case analytic sample of *N* = 222 (eight patients had missing HbA1c values); all model-derived statistics, including regression coefficients, adjusted odds ratios, and the supplementary model diagnostics, were computed from this fitted model and are reported on the *N* = 222 sample consistently throughout the manuscript and Supplementary Materials. The primary outcome was amputation level, categorized as major amputation (at or proximal to the ankle joint, including ankle disarticulation) or minor amputation (distal to the ankle joint, including toe, ray, and transmetatarsal procedures as recorded in the source record). Candidate explanatory variables encompassed demographic characteristics, diabetes type and duration, treatment characteristics, comorbidities, laboratory indices, and wound-related features.

The use of several record sources allowed the abstraction team to reconcile information recorded in different parts of the clinical pathway. Operative reports were used to identify procedure level, while admission records, nursing documentation, consultation notes, discharge summaries, and laboratory records supplied the explanatory variables. Re-abstraction of a random 10% sample evaluated consistency of the extraction process and yielded agreement above 95%. This quality-control result indicates reproducibility between abstractors for the sampled records; it does not demonstrate that the underlying routine documentation was complete or that every recorded diagnosis had been assessed with a uniform clinical test.

### 2.5. Operational Definitions of Key Variables

Peripheral neuropathy was defined as documentation in the medical record of a clinical diagnosis of diabetic peripheral neuropathy, a documented abnormal monofilament or vibration-perception examination, or a specialist diagnosis recorded in the neurology or endocrinology consultation notes. Peripheral arterial disease (PAD) was defined as documentation of a clinical diagnosis of PAD, an ankle–brachial index (ABI) or toe–brachial index (TBI) below the laboratory reference threshold, or an abnormal arterial duplex or angiographic finding recorded in the vascular surgery or radiology notes; because formal vascular measurements were not consistently available in the retrospective record, PAD ascertainment was heterogeneous, and this limitation is addressed in the Discussion. Multiple foot ulcers was defined as the presence of two or more concurrent foot ulcers documented on admission examination; ulcer duration >1 month was defined as a documented ulcer present for more than one month before admission. Illiteracy was defined as the patient’s documented inability to read or write as recorded in the admission or nursing documentation. Insulin therapy was defined as current insulin-based treatment at the time of admission. Comorbid conditions were abstracted individually from the medical record using documented diagnoses and are presented separately in Table 1. Information on revascularization procedures (open surgical bypass, endovascular intervention, or classification as having no revascularization option) and on the outcome of any revascularization relative to amputation level was not consistently recorded in the source records and could therefore not be captured in the analysis; this is acknowledged as a limitation. Infection was defined as the presence of cellulitis or osteomyelitis documented in the medical record, and this composite variable was used for the interaction term testing.

These operational definitions intentionally describe recorded information. A patient classified as not having documented neuropathy or PAD may have had no disease, an unrecorded assessment, or an assessment performed with a method not recoverable from the chart. Likewise, ulcer number and duration depended on the admission history and examination available to the abstraction team. The resulting variables should therefore be interpreted as documented clinical characteristics rather than direct measures of the underlying biological prevalence or severity of neuropathy, ischemia, and tissue loss. This distinction is particularly important when comparing effect estimates across institutions with different documentation and diagnostic practices.

### 2.6. Statistical Analysis

Data were analyzed using IBM SPSS Statistics for Windows, Version 27.0 (IBM Corp., Armonk, NY, USA) and R Version 4.2.0 (R Foundation for Statistical Computing, Vienna, Austria). Continuous variables were summarized as mean ± standard deviation when approximately normally distributed and as median with interquartile range when skewed; categorical variables were described as frequencies and percentages. Bivariate comparisons between the major and minor amputation groups were performed using independent-samples *t*-tests or Mann–Whitney U tests for continuous variables and Pearson’s chi-square or Fisher’s exact tests for categorical variables, as appropriate. Unadjusted odds ratios (ORs) with 95% confidence intervals (CIs) were reported. A two-sided *p*-value < 0.05 was considered statistically significant. These descriptive and bivariate analyses were conducted on the full cohort (*N* = 230).

To examine factors independently associated with major amputation, a multivariable binary logistic regression model was fitted. All candidate variables were specified before model fitting on the basis of subject-matter knowledge and their hypothesized roles in neuropathic, vascular, and wound-severity pathways relevant to amputation level; automated stepwise selection was not used. The prespecified candidate set comprised age, sex, diabetes duration, HbA1c, insulin therapy, illiteracy, previous foot ulcer, previous amputation, hypertension, ischemic heart disease, peripheral arterial disease, peripheral neuropathy, multiple foot ulcers, and ulcer duration >1 month. From this prespecified candidate set, the final adjustment set of six variables—documented peripheral neuropathy, IHD, multiple foot ulcers (≥2), ulcer duration >1 month, age (per 5 years), and HbA1c (per %)—was selected a priori because each was hypothesized to capture neuropathic, vascular, or wound-severity pathways relevant to amputation level. Bivariate screening (*p* < 0.10) was used only as a supplementary, exploratory aid to variable selection; this criterion is explicitly acknowledged as a data-driven, exploratory approach, it does not constitute a method for identifying confounders, and the resulting adjusted associations should therefore be interpreted as exploratory. The variable selection step was not repeated within each bootstrap resample used for the supplementary diagnostics. Continuous variables, including age and HbA1c, were assessed for non-linearity with restricted cubic splines and were modeled linearly because non-linearity was not statistically significant. Multicollinearity was evaluated with the variance inflation factor (VIF), and tested clinical interactions (PAD × neuropathy, IHD × PAD, and ulcer duration × infection) were not statistically significant. For the interaction testing, infection was defined as the presence of documented cellulitis or osteomyelitis. Because peripheral neuropathy was infrequent, Firth’s penalized logistic regression was used to reduce small-sample bias and mitigate separation [16]. This was implemented using the ‘logistf’ package in R, with 95% confidence intervals derived from profile penalized likelihood and *p*-values from Wald tests. All post-estimation statistics, including VIF and the supplementary model diagnostics described below, were computed from this penalized model fit. The adjustment set was based on subject-matter knowledge and clinical rationale rather than on a formal causal framework (e.g., a directed acyclic graph); the absence of an explicit causal framework is acknowledged as a limitation, and residual confounding cannot be excluded.

**Figure 1:**
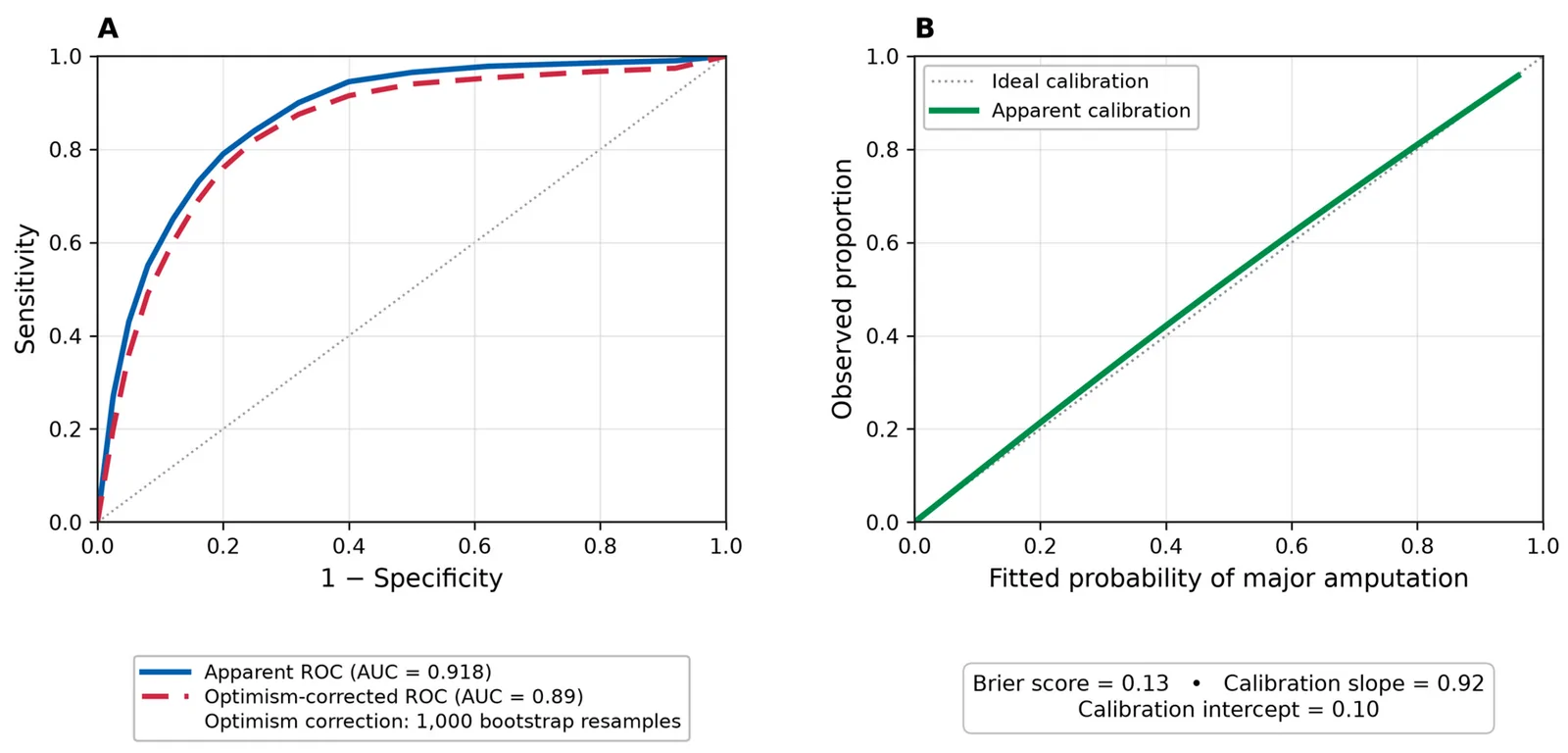
Exploratory model diagnostics for the fitted multivariable model. (**A**) Receiver operating characteristic curve, with apparent area under the curve (AUC) of 0.918 and optimism-corrected AUC of 0.89 (1000 bootstrap resamples). (**B**) Calibration plot showing agreement between observed and fitted probabilities of major lower extremity amputation, with a Brier score of 0.13, calibration slope of 0.92, and intercept of 0.10. Curve shapes are shown schematically and aligned to the summary diagnostics reported in the manuscript and Supplementary Table S4. These statistics quantify the internal fit of the explanatory model within this cohort and are not measures of predictive performance, diagnostic accuracy, or clinical utility [17].

Supplementary model diagnostics. Because the objective of the analysis is explanatory, the principal results are the adjusted odds ratios with their 95% confidence intervals. To document the internal stability of the fitted model, exploratory discrimination and calibration statistics were computed from the same penalized fit in the complete-case analytic sample (N = 222). The results are presented in Figure 1, with corresponding numerical values in Supplementary Table S4: the apparent area under the receiver operating characteristic curve (AUC), the optimism-corrected AUC based on 1,000 bootstrap resamples, the Hosmer–Lemeshow goodness-of-fit test, Nagelkerke R^2^, the calibration intercept, the calibration slope, and the Brier score. For each bootstrap resample, the model was refitted and the difference between the apparent and test AUC (optimism) was calculated; mean optimism was then subtracted from the apparent AUC to obtain the optimism-corrected AUC. Variable selection was not repeated within the bootstrap resamples, and a 95% confidence interval for the optimism-corrected AUC was not calculated; both are acknowledged limitations of these supplementary diagnostics. These statistics quantify the internal fit of the explanatory model within this cohort. They are not measures of predictive performance and do not establish diagnostic or clinical utility; explanatory and predictive modeling have distinct analytical goals [17].

In the fitted model, each aOR represents the relative odds of major versus minor LEA associated with that variable while holding the other listed model terms constant. The 95% CI describes the precision of the estimated association; wide intervals indicate that materially different effect magnitudes remain compatible with the data. Firth’s penalization reduces finite-sample bias and instability arising from sparse cells or separation, but it cannot correct misclassification of recorded predictors, selection into the amputated cohort, omitted variables, or residual confounding. The adjusted estimates were consequently interpreted as conditional associations rather than causal effects.

## 3. Results

### 3.1. Baseline Characteristics

The cohort comprised 230 hospitalized patients with diabetes who underwent LEA. Major amputation was performed in 92 patients (40.0%), whereas 138 patients (60.0%) underwent minor amputation. The mean age was 58.4 ± 12.6 years, 77.0% of participants were male, and 87.0% were Saudi nationals. Type 2 diabetes was present in 83.5% of the cohort, 80.9% had diabetes duration >10 years, and mean HbA1c was 10.6 ± 2.1%. Insulin therapy and higher HbA1c were more common in the major amputation group in unadjusted comparisons (Table 1). Additional candidate predictors, including BMI, Wagner grade, serum albumin, creatinine, white blood cell count, osteomyelitis, cellulitis, and smoking status, were evaluated but were not statistically significant in bivariate analysis (Supplementary Table S3).

Taken together, these characteristics describe a predominantly male hospital cohort with longstanding diabetes, poor average glycemic control, and established operative limb loss. They should not be read as prevalence estimates for people with diabetes in Makkah or Saudi Arabia because the study did not sample the general diabetes population or all patients with DFU. The profile instead defines the clinical spectrum within which the major-versus-minor comparison was made and provides the denominator needed to interpret the subsequent bivariate and adjusted associations.

**Table 1:** Baseline demographic, clinical, and comorbid characteristics of the study population (N = 230).

Characteristic	Total (N = 230)	Minor (n = 138)	Major (n = 92)	*p* Value
Demographics				
Age, years	58.4 ± 12.6	57.1 ± 13.1	60.4 ± 11.7	0.056
Male sex, *n* (%)	177 (77.0)	105 (76.1)	72 (78.3)	0.742
Saudi nationality, *n* (%)	200 (87.0)	122 (88.4)	78 (84.8)	0.432
Smoker (current or former), *n* (%)	148 (64.3)	86 (62.3)	62 (67.4)	0.568
Diabetes profile				
Type 2 diabetes, n (%)	192 (83.5)	116 (84.1)	76 (82.6)	0.765
Duration > 10 years, n (%)	186 (80.9)	108 (78.3)	78 (84.8)	0.221
Insulin therapy, n (%)	154 (67.0)	79 (57.2)	75 (81.5)	<0.001
HbA1c, %	10.6 ± 2.1	10.2 ± 2.2	11.2 ± 1.7	0.004
HbA1c, mmol/mol	92 ± 23	88 ± 24	99 ± 19	—
Comorbid conditions, *n* (%)				
Hypertension	176 (76.5)	130 (94.2)	46 (50.0)	—
Ischemic heart disease	105 (45.7)	36 (26.1)	69 (75.0)	—
Diabetic retinopathy	100 (43.5)	62 (44.9)	38 (41.3)	—
Cellulitis	95 (41.3)	49 (35.5)	46 (50.0)	—
Osteomyelitis	94 (40.9)	52 (37.7)	42 (45.7)	—
Peripheral arterial disease	92 (40.0)	35 (25.4)	57 (62.0)	—
Anemia	43 (18.7)	26 (18.8)	17 (18.5)	—
Acute myocardial infarction	41 (17.8)	22 (15.9)	19 (20.7)	—
Chronic kidney disease	31 (13.5)	19 (13.8)	12 (13.0)	—
Peripheral neuropathy	28 (12.2)	5 (3.6)	23 (25.0)	—
Cerebrovascular accident	21 (9.1)	13 (9.4)	8 (8.7)	—
Charcot arthropathy	18 (7.8)	11 (8.0)	7 (7.6)	—

Data are mean ± SD or n (%) unless otherwise indicated. Bold values indicate statistical significance (*p* < 0.05). HbA1c in mmol/mol is calculated from the DCCT-aligned percentage. *p* values for categorical variables were calculated using the χ^2^ test or Fisher’s exact test; *p* values for continuous variables were calculated using the independent *t*-test (with Welch correction applied for unequal variances, as verified for HbA1c). Group-specific frequencies and percentages for each comorbid condition (minor versus major amputation columns) are now provided; because between-group comparisons for these comorbidities are reported in Table 2, no *p* values are displayed in this table. Denominators for all calculations reflect the complete-case sample for each respective variable (e.g., HbA1c n = 222 due to 3.5% missingness). Abbreviations: HbA1c, glycated hemoglobin; SD, standard deviation.

### 3.2. Comorbidity Burden

Comorbidity burden was high across the cohort (Table 1). Hypertension was the most common comorbidity (76.5%), followed by IHD (45.7%) and diabetic retinopathy (43.5%). PAD was documented in 40.0% of patients. Peripheral neuropathy was documented in only 12.2% of records, a proportion that is lower than would typically be expected in advanced diabetic foot disease and that may reflect incomplete ascertainment in routine clinical documentation. Osteomyelitis and cellulitis were each present in approximately two-fifths of the cohort. Comorbid conditions are presented individually in Table 1 to permit direct comparison of the documented disease burden across the cohort.

The contrast between the high frequency of recorded cardiovascular and infectious comorbidity and the low frequency of documented neuropathy is relevant to interpretation. It may reflect true case-mix differences, incomplete sensory examination, incomplete transfer of outpatient findings into the inpatient record, or a combination of these mechanisms. The retrospective data cannot distinguish among them. Accordingly, the 12.2% value describes chart documentation in this cohort and should not be used as an estimate of neuropathy prevalence among patients with advanced diabetic foot disease.

### 3.3. Bivariate Analysis

Several variables differed substantially between patients who underwent major and minor amputation (Table 2). Major amputation was more frequent among patients receiving insulin therapy, among those with a history of previous foot ulcers, and among those with prior amputation. Illiteracy was also more common in the major amputation group. Among comorbidities, documented peripheral neuropathy showed the strongest unadjusted association with major amputation (OR 9.00, 95% CI 3.30–24.50; *p* < 0.001), although the estimate was based on only 28 documented cases. IHD (OR 8.45, 95% CI 4.50–15.80; *p* < 0.001), PAD (OR 5.00, 95% CI 2.85–8.77; *p* < 0.001), and hypertension (OR 3.95, 95% CI 1.85–8.44; *p* = 0.007) were also more frequent in the major amputation group. Age did not reach conventional statistical significance in bivariate analysis (*p* = 0.056).

**Table 2:** Bivariate analysis of factors associated with amputation level.

Variable	Minor (n = 138)	Major (n = 92)	Unadjusted OR (95% CI)	*p* Value
Illiterate education	13 (9.4)	30 (32.6)	4.68 (2.29–9.58)	0.003
Previous foot ulcer	84 (60.9)	82 (89.1)	5.21 (2.55–10.63)	<0.001
Previous amputation	62 (44.9)	74 (80.4)	4.97 (2.73–9.05)	<0.001
Peripheral neuropathy	5 (3.6)	23 (25.0)	9.00 (3.30–24.50)	<0.001
Ischemic heart disease	36 (26.1)	69 (75.0)	8.45 (4.50–15.80)	<0.001
Peripheral arterial disease	35 (25.4)	57 (62.0)	5.00 (2.85–8.77)	<0.001
Hypertension	94 (67.8)	82 (89.3)	3.95 (1.85–8.44)	0.007

Note. OR: odds ratio; CI: confidence interval. All displayed values were rigorously re-verified and regenerated from a single unified analytic dataset (N = 230). The bivariate counts for each variable strictly correspond to the total frequencies reported in Table 1. Unadjusted odds ratios (ORs) and 95% confidence intervals (CIs) were calculated using exact binomial or asymptotic methods as appropriate for cell sizes. *p* values were derived from the chi-square test or Fisher’s exact test based on the final analytic dataset. Additional evaluated variables (BMI, Wagner grade, albumin, creatinine, WBC, osteomyelitis, cellulitis, smoking) were not significant and are reported in Supplementary Table S3.

### 3.4. Multivariable Analysis

After adjustment for the prespecified covariates, four variables remained independently associated with major amputation (Table 3). Documented peripheral neuropathy was associated with higher odds of major amputation (aOR 7.46, 95% CI 1.13–49.35; *p* = 0.037), but the wide confidence interval indicates substantial imprecision. IHD was also independently associated with major amputation (aOR 5.20, 95% CI 1.63–16.55; *p* = 0.005). Two wound-related characteristics remained significant in the final model: multiple foot ulcers (aOR 4.65, 95% CI 1.53–14.17; *p* = 0.007) and ulcer duration >1 month (aOR 3.15, 95% CI 1.09–9.10; *p* = 0.034). Age and HbA1c were retained for clinical adjustment but were not statistically significant independent correlates. PAD demonstrated a strong unadjusted association with major amputation but was not retained in the final model. Given the low VIF values, this attenuation likely reflects overlap with related vascular and wound-severity variables rather than unstable estimation from severe multicollinearity alone, and the pattern should be interpreted as evidence of shared clinical information across covariates rather than absence of vascular contribution (Supplementary Table S2). The neuropathy estimate in particular should be regarded as highly uncertain: the uncertainty arises primarily from the small number of patients with documented peripheral neuropathy (n = 28 in the cohort, of whom 23 underwent major amputation), which produces sparse data and a very wide confidence interval for the estimated association, rather than from missing data for this variable, which were few (Supplementary Table S1). Its external validity remains unconfirmed.

**Table 3:** Factors independently associated with major amputation.

Predictor Variable	B	S.E.	Wald	VIF	aOR (95% CI)	*p* Value
Peripheral neuropathy	2.01	0.963	4.36	1.12	7.46 (1.13–49.35)	0.037
Ischemic heart disease	1.649	0.591	7.79	1.25	5.20 (1.63–16.55)	0.005
Multiple foot ulcers (≥2)	1.537	0.568	7.32	1.18	4.65 (1.53–14.17)	0.007
Ulcer duration >1 month	1.147	0.541	4.49	1.09	3.15 (1.09–9.10)	0.034
Age (per 5 years)	0.225	0.14	2.58	1.15	1.25 (0.95–1.65)	0.108
HbA1c (per %)	0.18	0.12	2.25	1.08	1.20 (0.95–1.51)	0.133
Constant	−8.45	2.1	16.19	—	—	<0.001

Note. Model statistics: Nagelkerke R^2^ = 0.639; Hosmer–Lemeshow χ^2^ = 7.47, *p* = 0.487; Brier score = 0.13; calibration slope = 0.92; calibration intercept = 0.10. aOR: adjusted odds ratio; VIF: variance inflation factor. The regression coefficients (B) are Firth-penalized maximum likelihood estimates; they are reported for transparency and replication and do not constitute an individual prediction equation. The multivariable model was fitted on the complete-case analytic sample (N = 222). Apparent and optimism-corrected AUC, the calibration plot, and the Brier score are shown in Figure 1 and detailed numerically in Supplementary Table S4.

The adjusted findings identify several distinct information domains. IHD represented systemic cardiovascular burden, whereas ulcer multiplicity and prolonged duration represented documented wound burden and chronicity. Their adjusted associations persisted after simultaneous inclusion of age, HbA1c, and documented neuropathy. The neuropathy point estimate was large, but its confidence interval was substantially wider than those of the other retained associations, limiting the precision with which its magnitude can be described. Age and HbA1c remained clinically motivated adjustment variables without statistically significant independent associations in the fitted model.

The change in the PAD estimate between the unadjusted and multivariable analyses is also informative but not mechanistic. Adjustment can attenuate an association when covariates capture overlapping clinical information, when measurements are imprecise, or when the effective contrast changes after simultaneous conditioning. These data do not identify mediation and do not show that limb ischemia was absent or clinically unimportant. The results support only the narrower conclusion that documented PAD did not add a retained independent association under the reported model specification.

## 4. Discussion

### 4.1. Principal Findings

In this retrospective cohort of hospitalized patients with diabetes who underwent LEA, IHD, multiple foot ulcers, ulcer duration >1 month, and documented peripheral neuropathy were independently associated with major rather than minor amputation. The major amputation proportion of 40.0% represents the proportion of major amputations among the 230 amputations included in this referral cohort; because no denominator representing the overall population of patients with diabetic foot disease was available, this figure should not be interpreted as evidence of the severity of the broader inpatient population. These findings should be interpreted in light of the specific study population. The present analysis does not address which patients with diabetes or DFU will progress to any amputation; rather, among patients who had already reached the point of operative limb loss, it identifies characteristics associated with a more proximal amputation level.

The principal contribution of the study is therefore not a rule for selecting an operative level, but a structured description of the clinical information that accompanied major rather than minor procedures in this setting. The findings span systemic vascular disease, local wound burden, chronicity, and documented sensory impairment. Their coexistence is consistent with the multifactorial nature of advanced diabetic foot disease, in which tissue viability and the feasibility of distal healing are evaluated against the patient’s broader health status. Because treatment decisions and preoperative investigations were not modeled, the observed associations cannot reconstruct the multidisciplinary reasoning that produced each procedure.

A cautious near-term application is improvement of documentation and referral review. Patients presenting with multiple or longstanding ulcers and coexisting cardiovascular disease may benefit from complete recording of sensory findings, perfusion assessments, infection extent, prior interventions, and the timing of specialist evaluation. This recommendation concerns the completeness of assessment in advanced presentations; it does not imply that the current coefficients should be used to determine amputation level. Operative decisions remain individualized and require direct evaluation of anatomy, perfusion, infection, tissue viability, function, and patient goals.

Ischemic heart disease and systemic vascular burden IHD was a consistent independent correlate of major amputation in the adjusted model. This finding is clinically plausible because IHD may indicate diffuse atherosclerotic burden extending to peripheral and pedal arterial beds, as well as greater overall cardiovascular frailty [18]. Prior studies have likewise linked cardiovascular comorbidity with more severe lower-extremity outcomes in diabetic foot disease [11,19,20]. In the current cohort, the association between IHD and major amputation should be interpreted as an association-based marker of systemic vascular disease burden rather than evidence of a direct causal pathway. This interpretation is compatible with the observed attenuation of PAD in the adjusted model: both variables capture overlapping vascular information, and the imprecision and incomplete ascertainment of documented PAD in this retrospective cohort may have further limited its independent contribution.

Wound burden, chronicity, and the interpretation of PAD Multiple ulcers and ulcer duration >1 month were also independently associated with major amputation. These variables likely reflect a greater burden of tissue loss, delayed presentation, and more advanced local disease. The persistence of wound-related variables in the final model supports the clinical relevance of ulcer burden and chronicity when amputation level is considered among patients already requiring surgery, and is consistent with previous work showing that chronic, extensive diabetic foot wounds are associated with more severe surgical outcomes [21,22,23]. PAD was strongly associated with major amputation in unadjusted analysis but did not remain in the final model. This result should not be interpreted as evidence that PAD is clinically unimportant. Instead, the effect of documented PAD may have overlapped with other variables capturing vascular compromise and wound severity, including IHD, ulcer multiplicity, and prolonged ulcer duration, and PAD ascertainment may have been heterogeneous because formal vascular measures such as ankle-brachial index and toe-brachial index were not consistently available in the retrospective record. In addition, information on the type of revascularization performed (open surgical bypass versus endovascular intervention) or on patients considered to have no revascularization option, together with the outcome of any revascularization relative to amputation level, was not consistently recorded in the source records and could not be incorporated. This limits the ability to distinguish patients with correctable ischemia from those with irreversible tissue loss, and the absence of an independent PAD effect in the adjusted model should therefore be interpreted cautiously rather than as evidence against a vascular contribution to amputation level.

### 4.2. Peripheral Neuropathy and Measurement Limitations

Documented peripheral neuropathy was independently associated with major amputation, but this estimate requires cautious interpretation. The confidence interval was wide, reflecting limited precision, and only 12.2% of the cohort had neuropathy documented in the chart. This proportion is substantially lower than that reported in comparable diabetic foot populations, suggesting that the study likely captured documented neuropathy rather than true neuropathy prevalence [4,19,24]. Because the estimate was derived from a small number of documented cases, it should be regarded as highly uncertain and in need of replication in independent cohorts before any clinical interpretation. The finding supports the clinical importance of neuropathy while underscoring the limitations of retrospective ascertainment. More systematic bedside assessment, including standardized monofilament testing and structured documentation, may improve both clinical care and future observational research [25].

### 4.3. Glycemic Control and Other Metabolic Variables

Mean HbA1c was high across the cohort, indicating poor glycemic control at presentation. Nevertheless, HbA1c was not independently associated with amputation level after adjustment. In this selected cohort of patients who had already progressed to operative limb loss, a single admission measure of recent glycemic exposure may have provided less separation between major and minor procedures than the recorded neurovascular and wound characteristics. This finding should not be interpreted to mean that glycemic management is unimportant to ulcer prevention, wound healing, or long-term diabetes care. It is specific to the conditional outcome examined here and to the variables available in the retrospective record. Earlier hospital-based work has identified HbA1c as an independent correlate of major amputation in a different diabetic foot gangrene cohort [22], illustrating how outcome definition, case mix, measurement, and treatment context may contribute to variation across studies.

### 4.4. Strengths and Limitations

This study has several strengths. It examines a clinically important question in a high-burden regional context, includes a relatively favorable events-per-variable ratio for the final model, and uses Firth’s penalized regression to address sparse data [16]. It also reports supplementary exploratory diagnostics of the fitted model, which strengthens methodological transparency.

Several limitations should also be emphasized. First, the retrospective design depends on the completeness and accuracy of routine documentation and limits control over measurement quality. Second, the study was conducted in a single tertiary referral center, which may restrict generalizability and may enrich the cohort for more severe presentations. Third, because only amputated patients were included, the findings apply to the distinction between major and minor amputation and should not be generalized as predictors of amputation occurrence among all patients with DFU. Fourth, neuropathy and PAD were based on retrospective documentation and may have been underascertained or inconsistently classified, creating a risk of information bias that may have affected the estimated associations; Firth’s penalization addresses small-sample bias and separation but does not compensate for limited information or imperfect measurement of predictors. Fifth, unmeasured confounding remains possible, including factors such as standardized vascular imaging severity, time to specialist assessment, and revascularization details. Sixth, the pattern and nature of missing data were not fully characterized; although variable-level missingness was low (ranging from 0.0% to 4.8%), complete-case analysis may introduce bias if missingness was not completely at random. Finally, the reported associations are exploratory: the covariate-selection strategy included a supplementary, data-driven bivariate screening criterion (*p* < 0.10) in addition to prespecified clinical rationale, and the resulting adjusted associations should be interpreted accordingly. In addition, because the analysis was explanatory in orientation and did not apply a formal causal framework, the adjustment set was based on clinical rationale rather than on a directed acyclic graph, and residual confounding cannot be excluded.

Conditioning the cohort on completed amputation also creates a distinct form of selection. Characteristics associated with reaching surgery may be distributed differently among patients who undergo a major procedure and those who undergo a minor procedure than among all patients with an incident DFU. Associations observed within this operative subgroup may therefore differ from associations estimated at first ulcer presentation or during longitudinal limb-salvage care. Referral patterns, treatment before transfer, the availability and timing of vascular assessment, and clinical thresholds for surgery may further influence the observed case mix, although their direction and magnitude cannot be evaluated from the available records.

### 4.5. Future Implications

Future work should prioritize prospective multicenter cohorts that include standardized assessment of neuropathy, perfusion, wound severity, and treatment pathways. Such cohorts should also systematically record the type of revascularization (open surgical versus endovascular) or the classification of patients as having no revascularization option, together with the outcome of any revascularization relative to amputation level, so that the relationship between vascular intervention and amputation level can be evaluated directly. The present exploratory associations require confirmation in independent cohorts before any clinical application can be considered. Comparative evaluation against established diabetic foot classification systems, such as WIfI or PEDIS, may further clarify the added value of the variables identified here [26]. The use of the Wagner classification in the present study was pragmatic and reflects its availability in the source records; contemporary systems that integrate ischemia and infection, such as the Society for Vascular Surgery Wound, Ischemia, and foot Infection (WIfI) classification, should be considered in future work to improve the characterization of limb threat. In addition, studies addressing barriers to early presentation and access to multidisciplinary diabetic foot care in Saudi Arabia may help contextualize the associations observed in this high-risk hospital cohort [27,28,29]. Formal application of a causal framework, such as a directed acyclic graph, in future analyses would strengthen the identification of confounders and the specification of adjustment sets.

A prospective study should define the time origin at first specialist assessment or hospital presentation and enroll consecutive patients across multiple centers, including those managed without LEA as well as those who undergo minor or major procedures. This design would permit separate estimation of progression to any amputation and, among amputated patients, the level of the procedure. A prespecified data dictionary should distinguish index from repeat or staged operations, specify the handling of bilateral procedures, and define ankle disarticulation consistently. Standardized sensory testing, ABI or TBI where interpretable, arterial waveform or imaging findings, wound location and number, wound dimensions, infection severity, osteomyelitis, renal function, nutritional markers, and prior ulcer or amputation history should be collected at the same clinically meaningful time point.

Treatment pathways should also be represented explicitly. Future datasets should capture the timing of vascular, surgical, infectious-disease, endocrinology, podiatry, and wound-care assessment; whether revascularization was considered; the type and timing of open or endovascular intervention; technical and clinical outcomes; antimicrobial and debridement strategies; and the reason a limb or distal segment was judged unsalvageable. Recording these variables would allow investigators to separate baseline disease burden from treatment received and to examine whether associations differ across care pathways. Linkage to rehabilitation, readmission, re-ulceration, re-amputation, cardiovascular events, and mortality would place amputation level within a patient-centered longitudinal outcome framework.

Future analyses should distinguish explanatory aims from prediction aims at the design stage. If a prediction model is pursued, candidate variables and functional forms should be chosen before fitting, missing data should be addressed with methods appropriate to their mechanism, and any selection procedure should be repeated within each resampling iteration. Validation should be performed temporally and geographically, followed by recalibration when needed and formal evaluation of net benefit and clinical impact. Comparison with established limb-threat classifications, including systems that integrate wound, ischemia, and infection, could determine whether a new model provides information beyond structured clinical assessment. Only after these stages should electronic decision support be considered.

Before such a tool is available, the most defensible application of the present results is operational. Institutions can use the identified domains to audit whether cardiovascular history, neuropathy assessment, perfusion testing, wound number and duration, infection severity, and prior interventions are documented consistently for patients with advanced diabetic foot disease. Multidisciplinary teams can use those audits to identify gaps in assessment or referral pathways. This use aligns with the observational evidence while avoiding unsupported conversion of association estimates into individual surgical recommendations.

## 5. Conclusions

Among hospitalized patients with diabetes who underwent lower extremity amputation, IHD, multiple ulcers, ulcer duration >1 month, and documented peripheral neuropathy were independently associated with major rather than minor amputation. These findings describe associations and do not imply causal relationships. The neuropathy estimate should be interpreted cautiously because of its imprecision and the likelihood of underdocumentation. Overall, the findings support the importance of integrated cardiovascular, neurovascular, and wound assessment in patients presenting with advanced diabetic foot disease, while remaining strictly association-based in interpretation. These exploratory, conditional associations are hypothesis-generating and require confirmation in independent, prospectively designed cohorts before any clinical application can be considered.

Accordingly, the present findings are best used to generate hypotheses, strengthen documentation priorities, and guide future research in comparable advanced diabetic-foot settings; they should not be used as a surgical decision rule, as an individual prognosis, or as an estimate of amputation incidence.

## Data Availability

The datasets generated and/or analyzed during the current study are available from the corresponding author upon reasonable request and subject to institutional and ethical requirements.

## Supplementary Materials

The following supporting information can be downloaded at: https://www.mdpi.com/article/10.3390/jcm15207946/s1, Supplementary Figure S1. Flow chart of the participants. Supplementary Table S1. Missing data matrix for variables included in the analysis. Supplementary Table S2. Multicollinearity diagnostics for the final multivariable model. Supplementary Table S3: Bivariate analysis of non-significant candidate predictors. Supplementary Table S4. Diagnostic Performance Characteristics of the Prediction Model.
