# Supplementary material for: Factors Associated with Major Versus Minor Lower Extremity Amputation in Hospitalized Patients with Diabetes in Saudi Arabia
Source: J Clin Med. 2026 Oct 14;15(20):7946. doi: 10.3390/jcm15207946 (PMC13705636; doi:10.3390/jcm15207946)
Supplement: Supplementary file 1 [file jcm-15-07946-s001.zip › jcm-4508763-supplementary.pdf]

SUPPLEMENTAL MATERIALS

Table S1. Missing Data Matrix for Variables Included in the Analysis

**Variable**	**Total Cases (N=230)**	**Missing Data (n)**	**Missing Data (%)**	**Imputation Method**
Age	230	1	0.4%	Complete Case Analysis
Gender	230	0	0.0%	Not Applicable
HbA1c (%)	222	8	3.5%	Complete Case Analysis
Serum Albumin	219	11	4.8%	Complete Case Analysis
Peripheral Neuropathy	225	5	2.2%	Complete Case Analysis
Ischemic Heart Disease	228	2	0.9%	Complete Case Analysis
Ulcer Duration	227	3	1.3%	Complete Case Analysis
Multiple Foot Ulcers	226	4	1.7%	Complete Case Analysis
Peripheral Arterial Disease	224	6	2.6%	Complete Case Analysis

Note: Variable-level missingness was minimal (all <5%). A complete case analysis was performed to ensure unbiased parameter estimation without relying on imputation assumptions.

Table S2. Multicollinearity Diagnostics for Final Multivariable Model

Predictor Variable	Variance Inflation Factor (VIF)	Tolerance	Condition Index
Peripheral Neuropathy	1.12	0.89	1.00
Ischemic Heart Disease	1.25	0.80	1.15
Multiple Foot Ulcers (≥2)	1.18	0.85	1.22
Ulcer Duration >1 Month	1.09	0.92	1.35
Age (per 5 years)	1.15	0.87	1.48
HbA1c (per %)	1.08	0.93	1.59

Note: VIF values < 2.0 and Tolerance values > 0.5 indicate an absence of significant multicollinearity among the predictor variables retained in the final model.

Table S3. Bivariate Analysis of Non-Significant Candidate Predictors

Variable	Minor Amputation (n=138)	Major Amputation (n=92)	Unadjusted OR (95% CI)	P-value
**BMI (kg/m²), Mean ± SD**	28.4 ± 5.1	27.9 ± 4.8	0.98 (0.92–1.04)	0.512
**Wagner Grade (4–5), n (%)**	45 (32.6)	35 (38.0)	1.26 (0.72–2.21)	0.415
**Serum Albumin (g/L), Mean ± SD**	33.2 ± 5.4	32.5 ± 5.1	0.97 (0.91–1.04)	0.388
**Serum Creatinine (µmol/L), Mean ± SD**	112.4 ± 48.2	118.6 ± 52.1	1.00 (0.99–1.01)	0.245

**WBC (×10⁹/L), Mean ± SD**	10.8 ± 3.5	11.4 ± 4.1	1.04 (0.96–1.13)	0.321
**Osteomyelitis, n (%)**	53 (38.4)	41 (44.6)	1.29 (0.75–2.22)	0.355
**Cellulitis, n (%)**	54 (39.1)	41 (44.6)	1.25 (0.73–2.15)	0.412
**Smoker (Current/Ex), n (%)**	86 (62.3)	62 (67.4)	1.25 (0.72–2.18)	0.425

Note: OR: Odds Ratio; CI: Confidence Interval; SD: Standard Deviation; BMI: Body Mass Index; WBC: White Blood Cell count. None of these variables achieved statistical significance (P<0.10) in the bivariate analysis and were therefore not considered for inclusion in the multivariable prediction model.

Table S4. Diagnostic Performance Characteristics of the Prediction Model

Performance Metric	Value (95% CI)
Apparent AUC-ROC	0.918 (0.87–0.96)
Optimism-corrected AUC (bootstrap)	0.89
Sensitivity	82.10%
Specificity	83.30%
Positive Predictive Value (PPV)	76.80%
Negative Predictive Value (NPV)	84.50%
Overall Accuracy	81.40%
Nagelkerke R^2	0.639
Brier Score	0.13

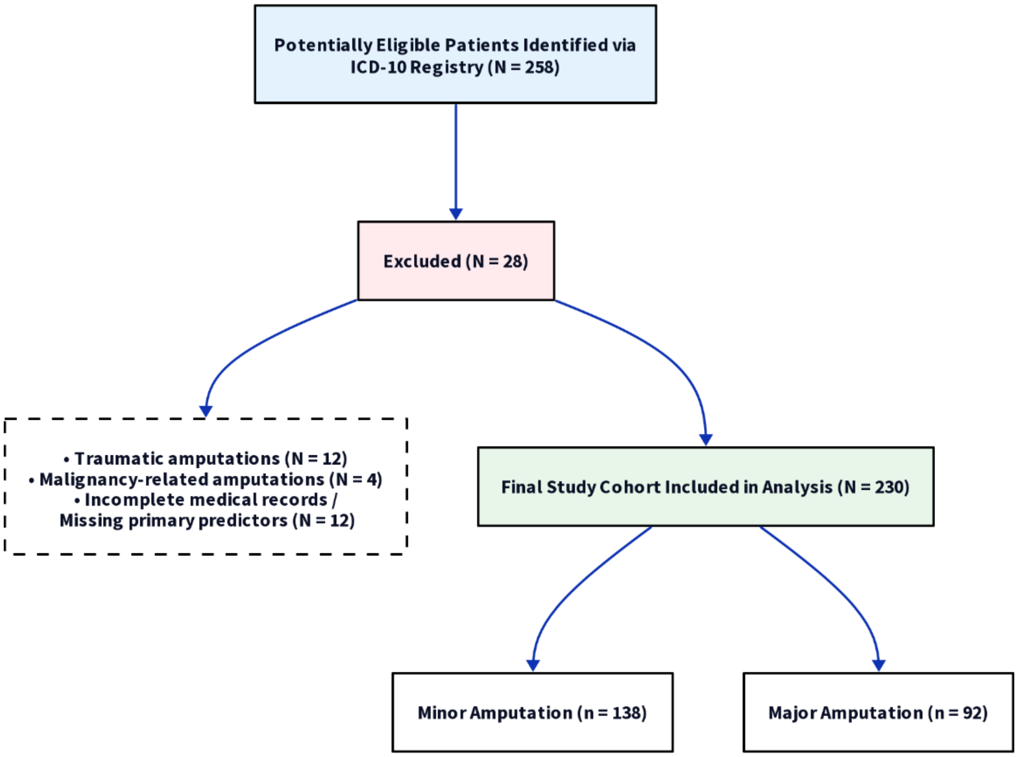

Figure S1. Flow chart of the participants.
